# Diffusion-weighted imaging-based radiomics model using automatic machine learning to differentiate cerebral cystic metastases from brain abscesses

**DOI:** 10.1007/s00432-024-05642-4

**Published:** 2024-03-16

**Authors:** Linyang Cui, Zheng Qin, Siyuan Sun, Weihua Feng, Mingyuan Hou, Dexin Yu

**Affiliations:** 1 Department of Radiology, Qilu Hospital of Shandong University, Jinan, 250012 Shandong China; 2 Department of Radiology, Weihai Central Hospital Affiliated to Qingdao University, Weihai, 264400 Shandong China; 3 Cheeloo College of Medicine, Shandong University, Jinan, 250012 Shandong China; 4 Qilu Pharmaceutical Co., Ltd, Jinan, 250100 Shandong China; 5 Department of Radiology, The Affiliated Hospital of Qingdao University, Qingdao, 266000 Shandong China; 6 Department of Imaging, The Affiliated Weihai Second Municipal Hospital of Qingdao University, Weihai, 264200 Shandong China

**Keywords:** Image normalization, Tree-based optimization tool, Cerebral cystic metastases, Brain abscesses

## Abstract

**Objectives:**

To develop a radiomics model based on diffusion-weighted imaging (DWI) utilizing automated machine learning method to differentiate cerebral cystic metastases from brain abscesses.

**Materials and methods:**

A total of 186 patients with cerebral cystic metastases (*n* = 98) and brain abscesses (*n* = 88) from two clinical institutions were retrospectively included. The datasets (129 from institution A) were randomly portioned into separate 75% training and 25% internal testing sets. Radiomics features were extracted from DWI images using two subregions of the lesion (cystic core and solid wall). A thorough image preprocessing method was applied to DWI images to ensure the robustness of radiomics features before feature extraction. Then the Tree-based Pipeline Optimization Tool (TPOT) was utilized to search for the best optimized machine learning pipeline, using a fivefold cross-validation in the training set. The external test set (57 from institution B) was used to evaluate the model’s performance.

**Results:**

Seven distinct TPOT models were optimized to distinguish between cerebral cystic metastases and abscesses either based on different features combination or using wavelet transform. The optimal model demonstrated an AUC of 1.00, an accuracy of 0.97, sensitivity of 1.00, and specificity of 0.93 in the internal test set, based on the combination of cystic core and solid wall radiomics signature using wavelet transform. In the external test set, this model reached 1.00 AUC, 0.96 accuracy, 1.00 sensitivity, and 0.93 specificity.

**Conclusion:**

The DWI-based radiomics model established by TPOT exhibits a promising predictive capacity in distinguishing cerebral cystic metastases from abscesses.

**Supplementary Information:**

The online version contains supplementary material available at 10.1007/s00432-024-05642-4.

## Introduction

Cerebral cystic metastases and abscesses present similar patterns on conventional magnetic resonance imaging (MRI), making it difficult to distinguish between them (Muccio et al. 2014). However, accurate differential diagnosis is crucial for appropriate clinical management due to the different prognoses and treatment options for each condition (Bodilsen et al. 2023; Aizer et al. 2022). Advanced MRI techniques may provide additional information to aid in distinguishing between these two entities (Lai et al. 2019; Martín-Noguerol et al. 2021; Falk Delgado et al. 2019). Among them, diffusion-weighted imaging (DWI) is the most used due to its accuracy and convenience. Brain abscesses typically exhibit markedly hyperintense signals in cavities with restricted diffusion of contents on DWI, while the cavities of cystic brain tumors generally show hypointense signal. However, some cystic brain metastases have been reported to present high intensity with low apparent diffusion coefficient (ADC) value on DWI because of highly viscous mucin or many inflammatory cells in the cystic cavity (Sakatani et al. 2019; Takayasu et al. 2018; Pérez-Riverola et al. 2023; Hartmann et al. 2001; Yikilmaz et al. 2009). In 2010, (Duygulu et al. 2010) reported that 19.7% of intracerebral metastasis showed hyperintensity for DWI in a larger patient cohort. Additionally, 5–21% of untreated abscesses display low DWI signal, mimicking necrotic tumors within the central portion (Reddy et al. 2006). In short, differentiation of cerebral cystic metastases from abscesses with DWI sometimes remains a challenge, and it is necessary to explorea more accurate and effective method.

Numerous studies have demonstrated that radiomics exhibit superior diagnostic capabilities compared to visual analysis in the diagnosis, classification, and outcome prediction of brain lesions (Rudie et al. 2019; Abdel Razek et al. 2021; Forghani 2020; Kalasauskas et al. 2022). Radiomics has the potential to better differentiate cerebral cystic metastases and abscesses. Although radiomics has advantage for various applications, several challenges still need to be addressed (Lohmann et al. 2022). One challenge is the issue of robustness, which arises due to the use of different image datasets. To address this, an image preprocessing pipeline has been proposed to overcome the problem of incomparability among datasets. In the past, machine learning required manual testing to select appropriate features and models, which was cumbersome and often relied heavily on human expertise. However, an automated machine learning tool has been developed to improve this process. The tree-based pipeline optimization tool (TPOT) is an example of a tool that can automatically optimize the best machine learning pipeline using genetic algorithms (Le et al. 2020). Recent studies have demonstrated TPOT’s superior ability to construct radiomic models, outperforming standard manual machine learning analysis (Peng et al. 2022; Zhang et al. 2021; Su et al. 2020; Radzi et al. 2021).

The aim of our study was to establish a radiomics model based on DWI with TPOT using dual-center MRI datasets and to evaluate its diagnostic accuracy in distinguishing cerebral cystic metastases from abscesses. Furthermore, we aimed to validate the reliability and resilience of our image preprocessing methodology in bolstering the validity of our conclusions.

## Materials and methods

### Patients

**Fig. 1:**
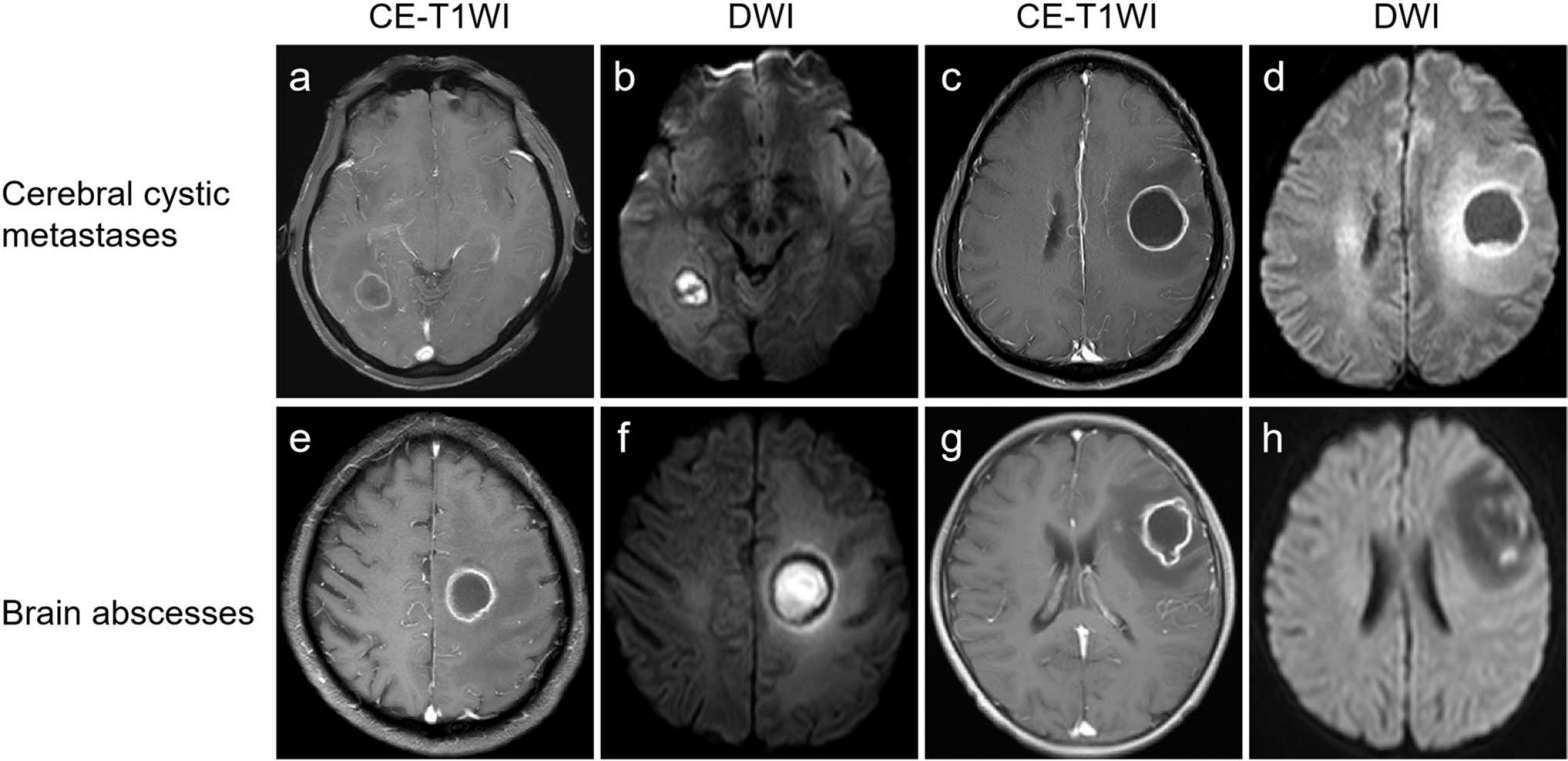
Representative examples of cerebral cystic metastases and brain abscesses of the capsular stage. Cerebral cystic metastases (**a**–**d**) and brain abscesses of the capsular stage (**e**–**h**) exhibit similarly rim enhancing on CE-T1WI. **a**, **b** A 51-year-old male with cerebral cystic metastasis from lung adenocarcinoma. The core of the lesion presented hyperintensity on DWI. **c**, **d** A 56-year-old male patient with cerebral cystic metastasis from esophageal carcinoma. The core of the lesion presented hypointensity on DWI. **e**, **f** A 58-year-old male with bacterial brain abscess. The core of the lesion presented hyperintensity on DWI. **g**, **h** A 9-year-old male patient with fungal brain abscess. The core of the lesion presented hypointensity on DWI

This retrospective study received approval from the institutional review board, and informed consent was waived. We searched the data of 382 patients with cerebral cystic metastases and brain abscesses identified by MRI on picture archiving and communication systems (PACS) from institution A and institution B between January 2012 and January 2021. The inclusion criteria were as follows: (1) cerebral cystic metastasis was confirmed by the pathological diagnosis of the primary tumor and clinical materials; brain abscess diagnosis depended on pathological findings and laboratory tests; (2) the pattern of cerebral cystic metastasis was solitary or multiple lesions appearing as rim-enhancing masses that were completely cystoid, namely enhancement wall and cystic fluid core; all cases of brain abscesses were in the capsule stage; (3) patients underwent plain and enhanced brain MRI scans before surgery or systemic medication. The exclusion criteria included: (1) lesion with a maximum diameter of less than 1 cm; (2) poor quality images; (3) large cystic with small nodular or partial cystic change in MRI scans depicting brain metastasis; abscess cavity containing air. Representative cases are shown in Fig. 1.

**Fig. 2:**
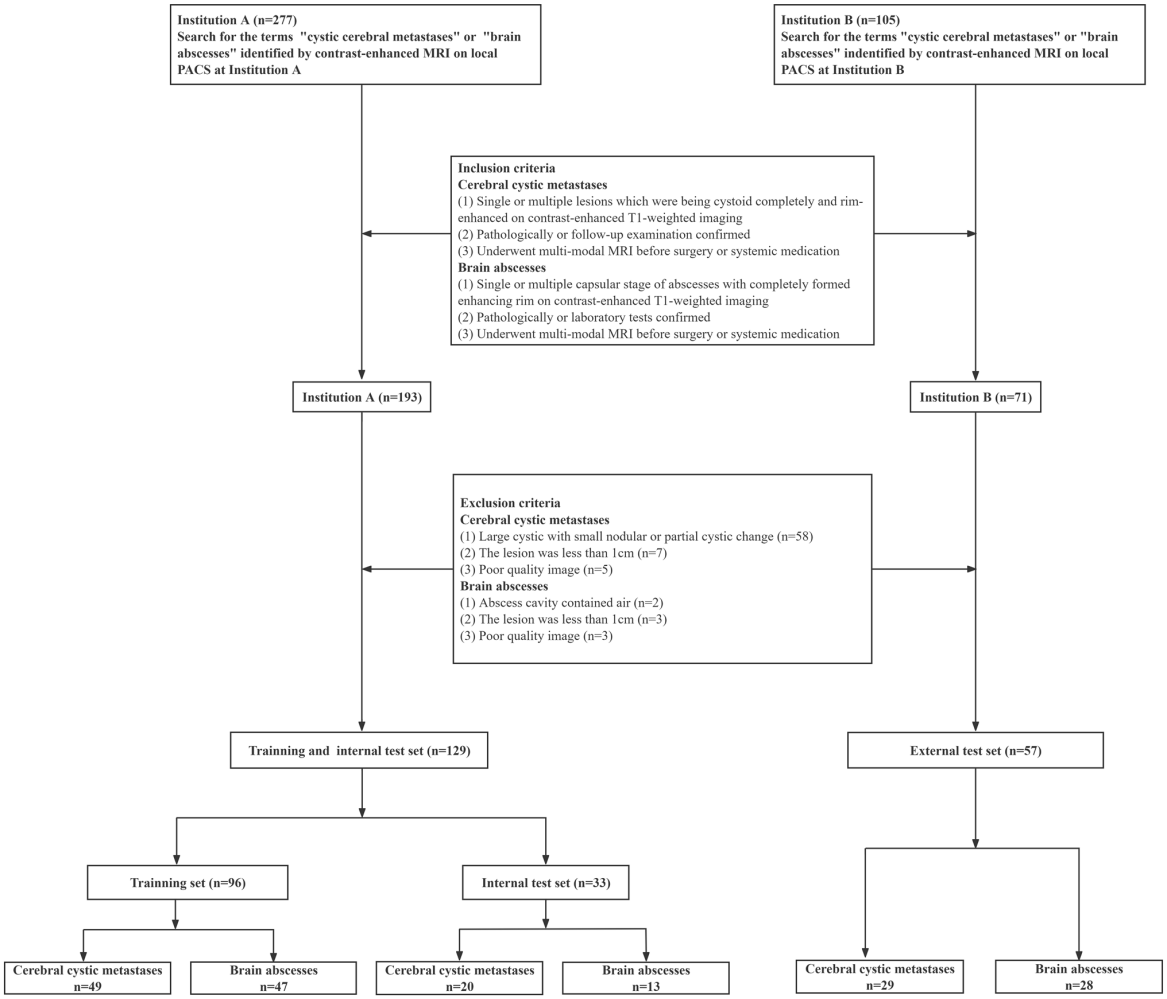
Study enrollment flowchart

This study involved a total of 186 patients who were diagnosed with either cerebral cystic metastases (*n* = 98) or brain abscesses (*n* = 88). Among the cases of cerebral cystic metastases, the primary tumors were identified as lung carcinoma (*n* = 87), esophageal carcinoma (*n* = 4), hepatic carcinoma (*n* = 2), renal carcinoma (*n* = 1), endometrial carcinoma (*n* = 1), breast carcinoma (*n* = 1), gastric carcinoma (*n* = 1), and rectal carcinoma (*n* = 1). The 88 patients with brain abscesses were categorized according to their pathogen, which included 64 bacterial, 8 fungal, 6 tubercular, 1 mixed infection of bacterial and fungal abscess, and 9 cases with unknown pathogens. The patients were divided into three groups: a training set (*n* = 96 patients) from institution A, an internal test set (*n* = 33 patients) from institution A, and an external test set (*n* = 57 patients) from institution B. The enrollment process for the study is depicted in Fig. 2.

### Image acquisition

MRI examinations were conducted at institution A using three imaging systems including Achieva 1.5 T and 3.0 T MRI scanner (Philips Healthcare, Best, The Netherlands), and Verio 3 T MRI scanner (Siemens Healthcare, Erlangen, Germany). The independent external data was gathered at institution B on the following MRI scanners: signa HDxt 1.5 T and signa HDX 3.0 T (GE Healthcare, Milwaukee, USA), Skyra 3 T (Siemens Healthcare, Erlangen, Germany). The scan sequence involved axial T2-weighted imaging (T2WI), T1-weighted imaging (T1WI), DWI, and contrast-enhanced T1WI (CE-T1WI). The DWI had *b* values of 0 and 1000 s/mm^2^, with the latter being used for analysis. The ADC maps were generated automatically by MRI scanners or manually reconstructed on the MRI scanner’s post-processing workstation. Please refer to Table 1 for detailed parameters.

**Table 1:** MR scan protocols

Scanner	Sequence	TR (ms)	TE (ms)	FOV (cm)	Slice thickness (mm)	Slice gap (mm)
Philips Achieva 3.0 T	T2WI	3000	80	23	6	1
	T1WI	2000	10	23	6	1
	DWI	3000	106	23	6	1
	CE-T1WI	194	4.6	23	6	1
Philips Achieva 1.5 T	T2WI	4339	120	23	6	1
	T1WI	488	15	23	6	1
	DWI	2003	70.7	23	6	1
	CE-T1WI	488	15	23	6	1
Siemens Verio 3.0 T	T2WI	4000	93	23	6	1
	T1WI	1900	8.5	23	6	1
	DWI	4500	100	23	5	1
	CE-T1WI	250	2.5	23	5	1
GE signa HDX 3.0 T	T2WI	3160	104.8	24	5	1
	T1WI	2761	8.7	24	5	1
	DWI	5100	74	24	5	1
	CE-T1WI	1792	14.4	24	5	1
GE signa HDxt 1.5 T	T2WI	3460	109	24	5	1
	T1WI	1900	8.5	24	5	1
	DWI	4600	82	24	5	1
	CE-T1W	1966	8.7	24	5	1
Siemens Skyra 3 T	T2WI	3400	109	24	5	1
	T1WI	1900	8.5	24	5	1
	DWI	3200	73	24	5	1
	CE-T1WI	2000	8.5	24	5	1

*TR* Repetition time; *TE* echo time; *FOV* field of view; *T2WI* T2-weighted imaging; *DWI* diffusion-weighted imaging; *CE-T1WI* contrast-enhanced T1-weighted imaging

### Clinical and conventional MR analysis

Two neuroradiologists (reader A and reader B), with 6 and 18 years of experience, respectively, independently reviewed all MRI scans. The radiologists were blinded to clinical and pathological data and reached a consensus. In cases where multiple lesions were present, analysis was based on the largest lesion. MRI features were assessed based on the following criteria: (1) location (lobe, basal ganglion and thalamus, brain stem, cerebellum, multiple); (2) presence of hypointense rims on T2-weighted images; (3) pattern of wall enhancement (smooth inner and outer walls, rough inner and smooth outer walls, smooth inner and rough outer walls, or rough inner and outer walls); (4) thickness of the enhancement wall (< 3 mm or ≥ 3 mm); (5) degree of edema (none, slight, or obvious); (6) ADC value of the wall; (7) ADC value of the core; and (8) maximum diameter of the mass. The degree of edema was divided into none, slight (less than 10 mm) and obvious (at or above10 mm), based on the classification suggested by Schoenegger for the glioblastoma (Schoenegger et al. 2009). ADC values were computed using the post-processing workstation. The region of interest (ROI) for the enhancement wall and cystic fluid core was delineated separately at the largest sectional area of the mass and its two adjacent layers on the ADC map. In cases where the mass was too small for the three layers, the ROI was outlined three times on the maximum cross section of the mass. Three ADC values for the core and wall were calculated by two neuroradiologists, and the average value was determined. The maximum diameters were measured independently on CE-T1WI, and the average was taken. Clinical features such as age, sex, presence or absence of fever, and leukocytosis were obtained from the medical records.

### Image annotation

The process of image segmentation was carried out using the open-source software 3D Slicer 4.11.0, based on DWI sequences. The 3D ROI was manually delineated slice by slice on the DWI images (*b* = 1000 s/mm^2^) to cover the core and wall, with reference to CE-T1WI, without any prior medical information. All manual segmentations were performed by reader A and the results were verified by reader B. To assess the intra- and inter-class correlation coefficient (ICC), reader A performed the segmentation of 30 randomly selected cases twice at the 3-month interval, and reader B independently performed the segmentation of 30 patients following the same procedure. Features with ICCs greater than 0.75 were selected for subsequent analysis.

### Image preprocess

**Fig. 3:**
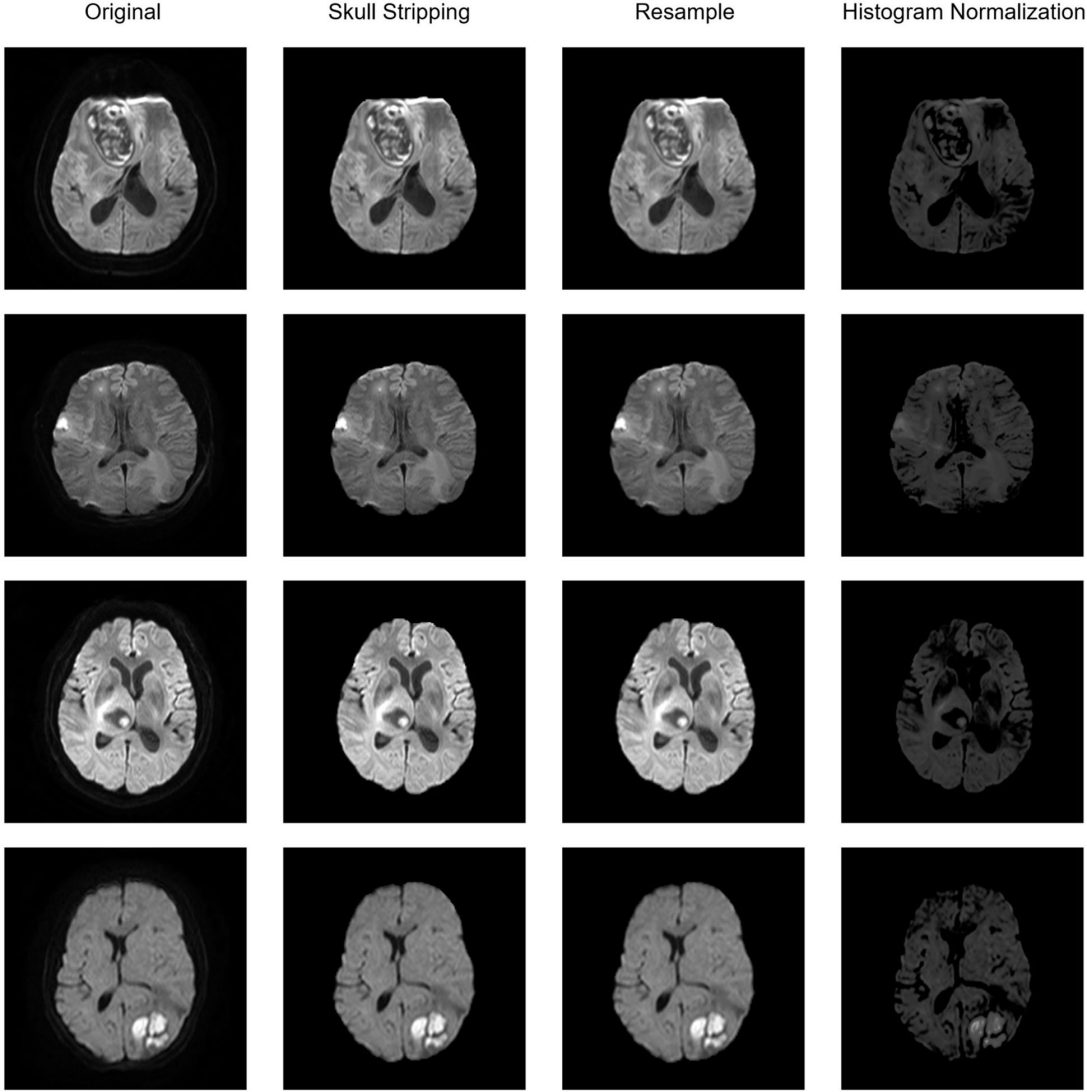
The pipeline of image preprocessing. The baseline preprocessing pipeline of four different cases are presented, including skull stripping, resample and histogram normalization

In this study conducted across two institutions, a thorough preprocessing method was developed for the analysis of various types of brain MR images. The method involved three steps: skull stripping to remove the skull, resampling to normalize spacing heterogeneity, and histogram normalization to reduce histogram distribution variance. The software HD-BET (Isensee et al. 2019), which is based on deep learning, was used to extract the brain and and strip the skull in the DWI sequence of MRI. The DWI images were then resampled to a consistent physical size of 1 mm, 1 mm, and 1 mm using Python SimpleITK package along with the simultaneous resampling of the mask of ROIs. Finally, the DWI sequences were normalized in histogram using a histogram match algorithm based on the feature of the histogram of a template collected from brain MRI image of a normal case in institution A. The pipeline of preprocessing is illustrated in Fig. 3. The importance of the DWI sequence preprocessing in radiomic model performance was further validated by comparing the baseline of the radiomic model before and after preprocessing.

### Feature extraction and automated clinical and radiomics model

**Fig. 4:**
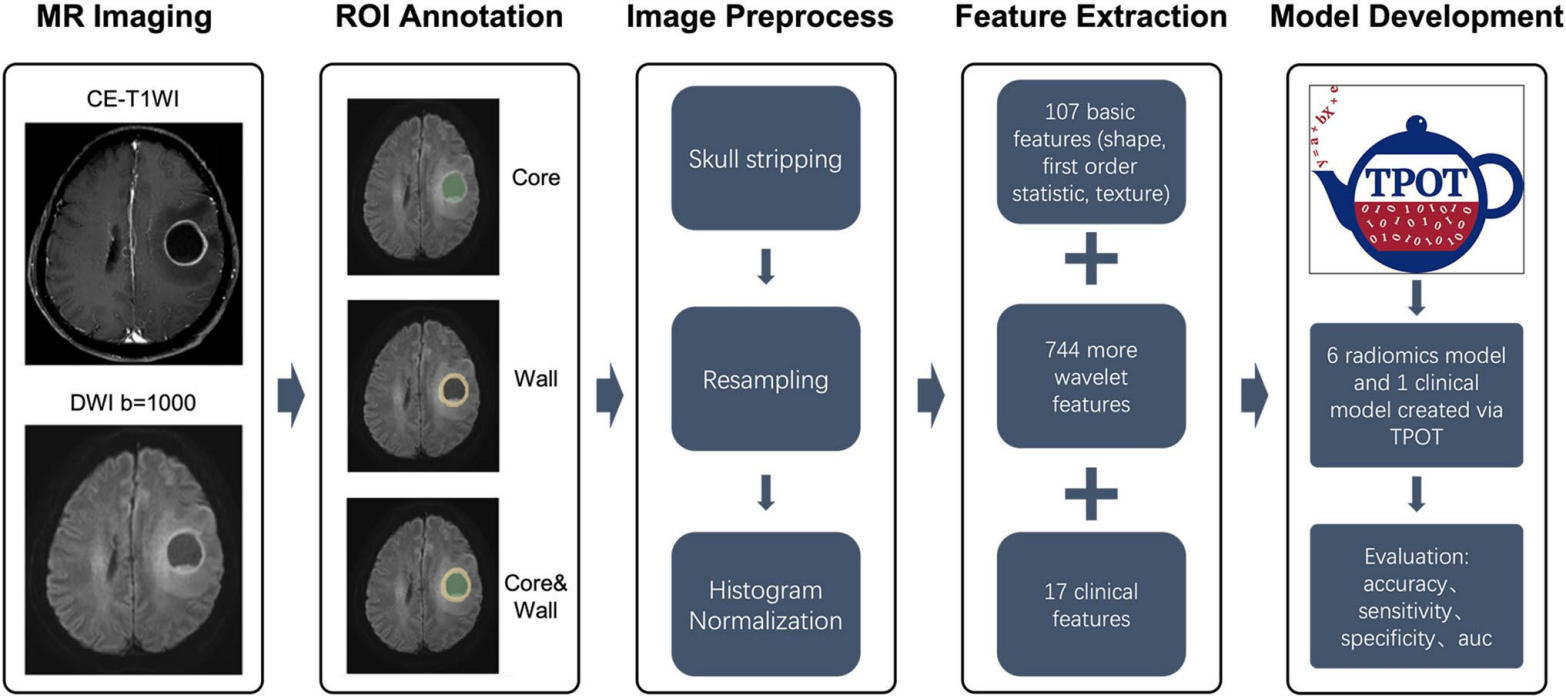
The workflow of this study

Radiomics features were extracted from two subregions on diffusion-weighted MR images, namely the cystic fluid core and solid wall (short: core and wall). Subregions (core, wall), individually or in combination, were assigned to three groups (core, wall, combination of core and wall). Combination of core and wall referred to extracting features from the core and wall, respectively, and subsequently combining these features. We compared the performance of the radiomic model using different combinations of features from different groups to identify the most significant features. TPOT is a Python-based automated machine learning tool for constructing radiomics and clinical models. During the training phase, the features extracted from the DWI sequence in the training dataset were fed into TPOT in Python to search for the optimal machine learning pipeline through fivefold cross-validation. Subsequently, the best machine learning pipeline was tested on the internal and external dataset to assess its generalizability. We placed equal importance on clinical and radiomic features. Thus, we conducted comparative experiments on both types of features using TPOT to identify the most significant machine learning pipeline. The model’s performance was evaluated by calculating the accuracy, sensitivity, specificity, and receiver operating characteristics area under the curve (ROC-AUC) values on the internal and external test dataset. DeLong’s test was used to compare the AUC value of clinical and all the radiomics models. The workflow of this study is shown in Fig. 4.

## Results

### Clinical characteristics

Table 2 presents the clinical and radiological characteristics of the cerebral cystic metastases and brain abscess groups. Our statistical analysis revealed significant differences (*p* < 0.05) in age leukocytosis, hypointense rims on T2WI, ADC value of the core, and ADC value of the wall between the two conditions on the training set. There were significant differences (*p* < 0.05) of fever, pattern of enhancement wall, and ADC value of the core between the two conditions in the internal test set. The factors including age, fever, location, hypointense rims on T2WI, pattern of enhancement wall, degree of edema, ADC value of the core, and the maximum diameter of the mass between the two conditions are statistically significant (*p* < 0.05) in the external test set.

**Table 2:** Clinical and radiological features in the training and test set

Characteristics	Training set (*n* = 96)		*p*	Internal test set (*n* = 33)		*p*	External test set (*n* = 57)		*p*
	Cerebral cystic metastases	Brain abscesses		Cerebral cystic metastases	Brain abscesses		Cerebral cystic metastases	Brain abscesses	
Age (years)	56.96 ± 11.15	46.04 ± 19.24	0.011*	54.70 ± 13.07	53.38 ± 14.68	0.788	62.69 ± 9.27	50.54 ± 16.83	< 0.001*
Sex									
Male	31 (63.27%)	31 (65.96%)	0.965	10 (50.00%)	8 (61.54%)	0.515	21 (72.42%)	24 (85.71%)	0.218
Female	18 (36.73%)	16 (34.04%)		10 (50.00%)	5 (38.46%)		8 (27.58%)	4 (14.29%)	
Fever									
Yes	10 (20.41%)	20 (42.55%)	0.578	4 (20.00%)	8 (61.54%)	0.015*	3 (10.34%)	15 (53.57%)	< 0.001*
No	39 (79.59%)	27 (57.45%)		16 (80.00%)	5 (38.46%)		26 (89.66%)	13 (46.43%)	
Leukocytosis									
Yes	3 (6.12%)	20 (42.55%)	0.004*	2 (10.00%)	5 (38.46%)	0.051	6 (20.69%)	12 (42.86%)	0.072
No	46 (93.88%)	27 (57.45%)		18 (90.00%)	8 (61.54%)		23 (79.31%)	16 (57.14%)	
Location									
Lobe	24 (48.98%)	31 (65.96%)	0.094	14 (70.00%)	8 (61.54%)	0.070	11 (37.93%)	21 (75.00%)	0.006*
Basal ganglion	2 (4.08%)	2 (4.26%)		0	0		0	1 (3.57%)	
Brain stem	1 (2.04%)	1 (2.13%)		0	0		0	0	
Cerebellum	3 (6.12%)	1 (2.13%)		0	2 (15.38%)		0	0	
Multiple	19 (38.78%)	12 (25.53%)		6 (30.00%)	3 (23.08%)		18 (62.07%)	6 (21.43%)	
Hypointense rims on T2WI									
Yes	8 (16.33%)	31 (65.96%)	0.001*	5 (25.00%)	5 (38.46%)	0.411	2 (6.90%)	9 (32.14%)	0.016*
No	41 (83.67%)	16 (34.04%)		15 (75.00%)	8 (61.54%)		27 (93.10%)	19 (67.86%)	
Pattern of enhancement wall									
Smooth inner and outer	9 (18.37%)	28 (59.57%)	0.080	6 (30.00%)	10 (76.92%)	< 0.001*	9 (31.03%)	14 (50.00%)	0.005*
Rough inner and smooth outer	33 (67.35%)	11 (23.40%)		13 (65.00%)	0		16 (55.17%)	4 (14.29%)	
Smooth inner and rough outer	0	4 (8.51%)		0	1 (7.69%)		0	4 (14.29%)	
Rough inner and outer	7 (14.29%)	4 (8.51%)		1 (5.00%)	2 (15.38%)		4 (13.79%)	6 (21.43%)	
The wall thickness									
< 3 mm	35 (71.43%)	29 (61.70%)	0.547	14 (70.00%)	10 (76.92%)	0.663	17 (58.62%)	12 (42.86%)	0.234
≥ 3 mm	14 (28.57%)	18 (38.30%)		6 (30.00%)	3 (23.08%)		12 (41.38%)	16 (57.14%)	
Degree of edema									
No	5 (10.20%)	0	0.138	0	0	0.900	1 (3.45%)	0	0.015*
Slight	17 (34.69%)	6 (12.77%)		5 (25.00%)	3 (23.08%)		12 (41.38%)	3 (10.71%)	
Obvious	27 (55.10%)	41 (87.23%)		15 (75.00%)	10 (76.92%)		16 (55.17%)	25 (89.29%)	
ADC value of the core (10^−6^ mm^2^/s)	1858.59 ± 694.07	836.32 ± 413.37	0.002*	2424.35 ± 333.43	594.23 ± 92.69	< 0.001*	2202.93 ± 674.85	709.57 ± 222.44	< 0.001*
ADC value of the wall (10^−6^ mm^2^/s)	1102.65 ± 236.27	1257.79 ± 256.81	0.025*	1260.50 ± 278.10	1275.62 ± 237.50	0.872	1255.86 ± 285.52	1311.89 ± 270.22	0.450
Maximum diameter of the mass (cm)	2.37 ± 1.13	2.85 ± 1.32	0.273	2.57 ± 0.96	2.36 ± 0.73	0.497	2.67 ± 1.36	3.43 ± 1.18	0.027*

*T2WI* T2-weighted imaging; *ADC* apparent diffusion coefficient

*Represents *p* < 0.05

### Image preprocessing

**Fig. 5:**
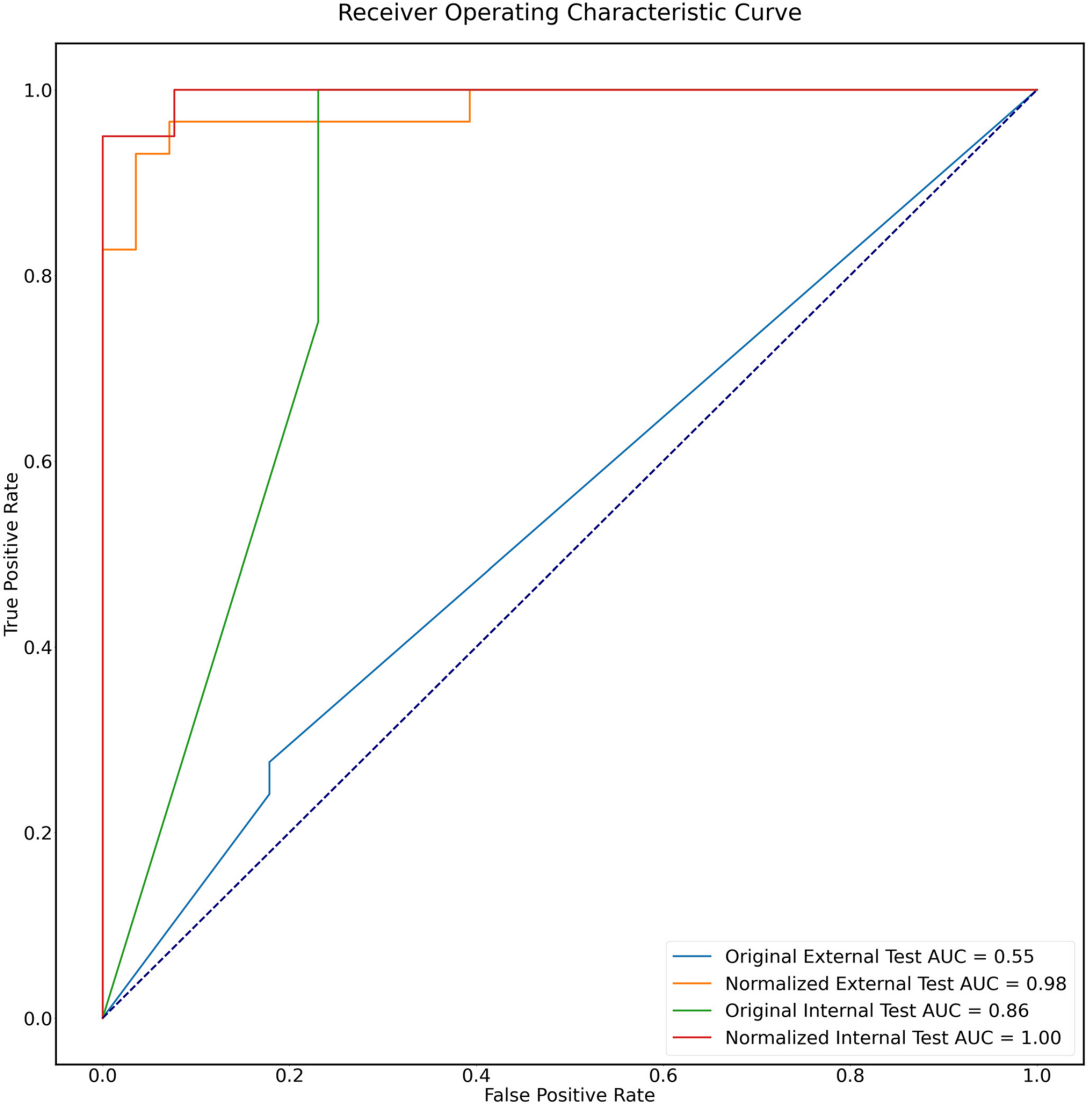
ROC curves of features’ performance before and after normalization in the internal and external test sets. The images normalized have better feature performance, compared to the original images both in the internal (AUC 1.00 vs. 0.86) and external test set (AUC 0.98 vs. 0.55)

The images normalized by a thorough preprocessing method have better feature performance, compared to the non-processed images both in the internal (AUC 1.00 vs. 0.86) and external test sets (AUC 0.98 vs. 0.55), as shown in Fig. 5.

### Feature extraction and automated model building

**Fig. 6:**
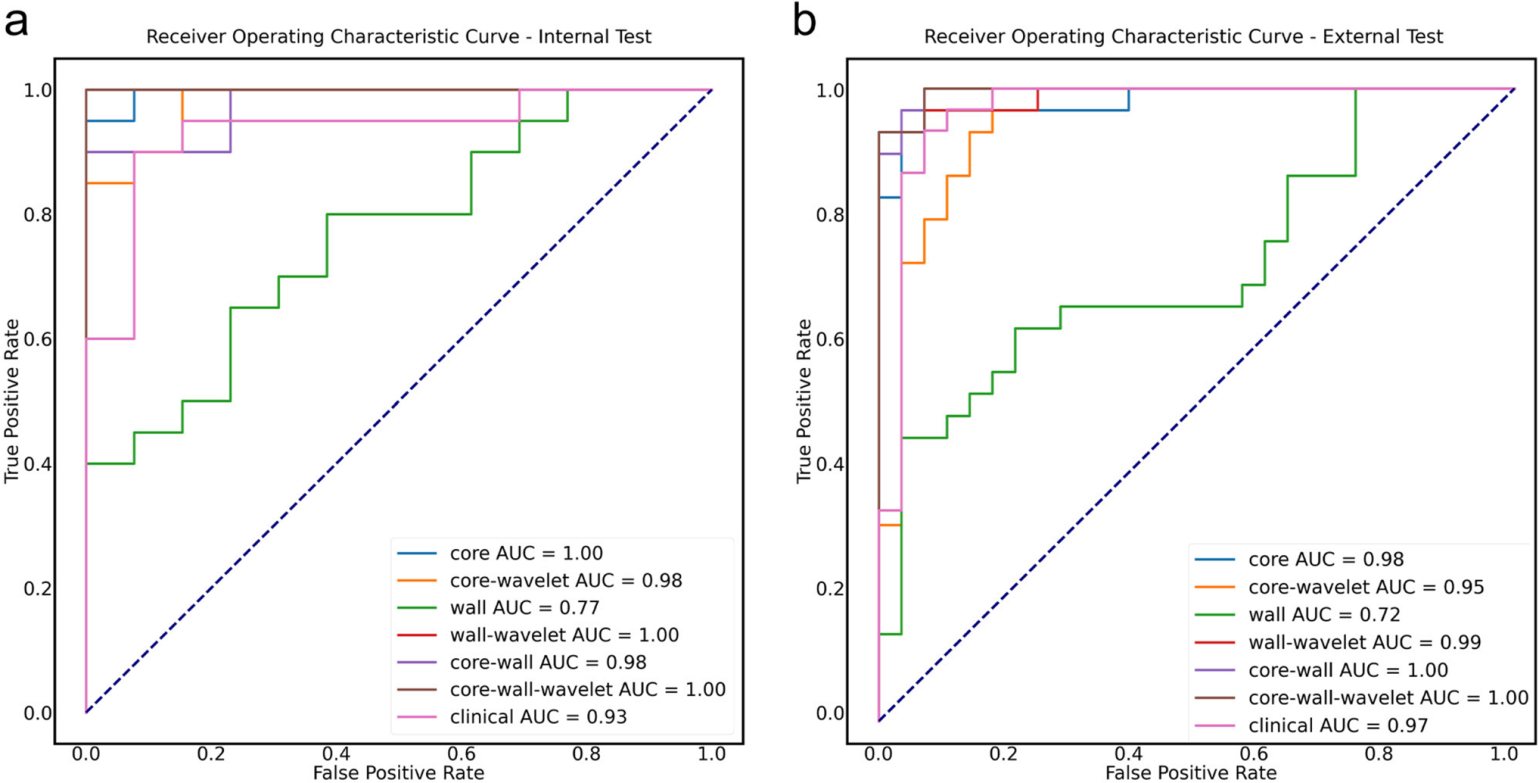
The ROC curves of seven distinct TPOT models. **a** The ROC curve of seven distinct TPOT models in the internal test set. **b** The ROC curve of seven distinct TPOT models in the external test set

**Fig. 7:**
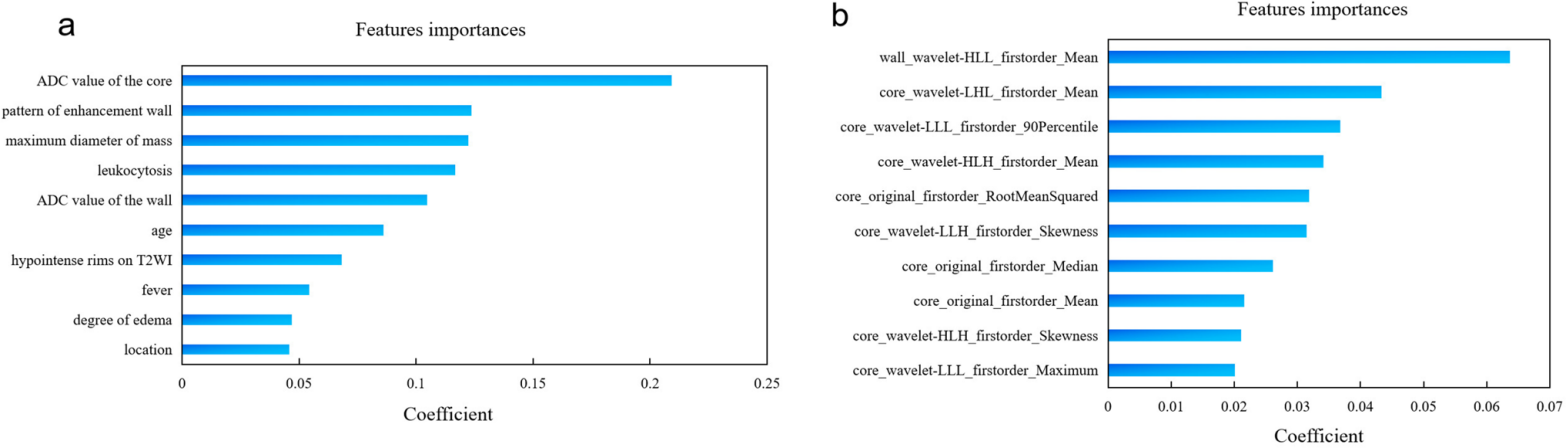
Coefficient of model’s features. **a** Coefficient of the clinical model’s top ten high-ranking features. **b** Coefficient of the best radiomics model’s top ten high-ranking radiomics features

On the manual segmentation, the intra- and inter-observer ICC values were 0.96 and 0.95, respectively, as shown in Supplemental Fig. 1. A total of 107 basic features, including first-order statistical features, shape features, and gray-level features were extracted after MR image preprocessing. To further enhance the model performance, we utilized wavelet filters to extract more subtle features, resulting in an additional 744 features. Seven TPOT models were created to distinguish cerebral cystic metastases from brain abscesses. Table 3 displays the classifiers and parameters for all the models. All prediction models performed reasonably well during training with the current best internal CV score greater than 0.80. With the exception of the radiomics model, based on the solid wall of lesion without wavelet transform, all TPOT models demonstrated excellent performance, with high accuracy and favorable AUC both on the internal and external test sets. The results including AUC, accuracy, sensitivity, and specificity of the different models are presented in Tables 4 and 5. The ROC curves of different models are shown in Fig. 6a, b. The clinical model’s AUC, accuracy, sensitivity, and specificity were 0.93, 0.88, 0.85, and 0.85, respectively, in the internal test set, and 0.97, 0.93, 0.93 and 0.93, respectively, in the external test set. The radiomics model based on the wavelet-transformed combination of core and wall features demonstrated the best performance with the highest AUCs of 1.00 both on the internal and external test sets. The optimal model demonstrated an accuracy of 0.97, sensitivity of 1.00, and specificity of 0.93 in the internal test sets and reached 0.96 accuracy, 1.00 sensitivity, and 0.93 specificity in the external test set. The clinical model’s top ten high-ranking features, in order, are the ADC value of core, the pattern of enhancement wall, the maximum diameter of mass, leukocytosis, ADC value of wall, age, hypointense rims on T2WI, fever, degree of edema, and location, as shown in Fig. 7a. The best model’s top ten high-ranking radiomics features are displayed in Fig. 7b, including the wall_wavelet-HLL_firstorder_Mean, core_wavelet-LHL_firstorder_Mean, core_wavelet-LLL_firstorder_90Percentile, core_wavelet-HLH_firstorder_Mean, core_original_firstorder_RootMeanSquared, core_wavelet-LLH_firstorder_Skewness, core_original_firstorder_Median, core_original_firstorder_Mean, core_wavelet-HLH_firstorder_Skewness, and core_wavelet-LLL_firstorder_Maximum.

**Table 3:** The classifiers and parameters of TPOT models

Model	Classifiers and parameters	Current best internal CV score
Clinical (baseline)	RandomForestClassifier(ZeroCount(input_matrix), bootstrap = False, criterion = gini, max_features = 0.05, min_samples_leaf = 14, min_samples_split = 2, n_estimators = 100)	0.9273
Core	ExtraTreesClassifier(input_matrix, bootstrap = False, criterion = entropy, max_features = 0.25, min_samples_leaf = 3, min_samples_split = 3, n_estimators = 100)	0.8647
Core wavelet	ExtraTreesClassifier(RobustScaler(input_matrix), bootstrap = False, criterion = entropy, max_features = 0.75, min_samples_leaf = 4, min_samples_split = 8, n_estimators = 100)	0.8853
Wall	GradientBoostingClassifier(input_matrix, learning_rate = 0.1, max_depth = 3, max_features = 0.30000000000000004, min_samples_leaf = 12, min_samples_split = 3, n_estimators = 100, subsample = 0.8500000000000001)	0.8026
Wall wavelet	ExtraTreesClassifier(CombineDFs(input_matrix, input_matrix), bootstrap = True, criterion = gini, max_features = 0.75, min_samples_leaf = 6, min_samples_split = 17, n_estimators = 100)	0.8742
Core wall	GradientBoostingClassifier(StandardScaler(GaussianNB(input_matrix)), learning_rate = 0.5, max_depth = 5, max_features = 0.9500000000000001, min_samples_leaf = 9, min_samples_split = 6, n_estimators = 100, subsample = 0.75)	0.8647
Core-wall wavelet	Best pipeline: ExtraTreesClassifier(input_matrix, bootstrap = True, criterion = entropy, max_features = 0.8500000000000001, min_samples_leaf = 4, min_samples_split = 15, n_estimators = 100)	0.8847

**Table 4:** TPOT models performance with the internal test dataset

Model	Accuracy (95% CI)	Sensitivity (95% CI)	Specificity (95% CI)	AUC (95% CI)	*p* value
Clinical (baseline)	0.88 (0.77–0.99)	0.85 (0.66–1.00)	0.85 (0.66–1.00)	0.93 (0.84–1.00)	/
Core	0.94 (0.86–1.00)	0.85 (0.66–1.00)	1.00 (1.00–1.00)	1.00 (0.99–1.00)	0.180
Core wavelet	0.88 (0.77–0.99)	0.92 (0.77–1.00)	0.80 (0.60–1.00)	0.98 (0.95–1.00)	0.358
Wall	0.7 (0.54–0.86)	0.62 (0.36–0.88)	0.62 (0.36–0.88)	0.77 (0.60–0.93)	0.080
Wall wavelet	0.97 (0.91–1.00)	1.00 (1.00–1.00)	0.93 (0.80–1.00)	1.00 (1.00–1.00)	0.147
Core wall	0.85 (0.73–0.97)	0.77 (0.54–1.00)	0.83 (0.62–1.00)	0.98 (0.94–1.00)	0.397
Core-wall wavelet	0.97 (0.91–1.00)	1.00 (1.00–1.00)	0.93 (0.80–1.00)	1.00 (1.00–1.00)	0.147

**Table 5:** TPOT model performance with the external test dataset

Model	Accuracy (95% CI)	Sensitivity (95% CI)	Specificity (95% CI)	AUC (95% CI)	*p* value
Clinical (baseline)	0.93 (0.86–1.00)	0.93 (0.84–1.00)	0.93 (0.84–1.00)	0.97 (0.92–1.00)	/
Core	0.95 (0.89–1.00)	0.93 (0.84–1.00)	0.96 (0.87–1.00)	0.98 (0.95–1.00)	0.613
Core wavelet	0.84 (0.74–0.94)	0.93 (0.84–1.00)	0.79 (0.65–0.93)	0.95 (0.89–1.00)	0.687
Wall	0.68 (0.56–0.80)	0.71 (0.54–0.88)	0.67 (0.50–0.84)	0.72 (0.58–0.85)	< 0.001*
Wall wavelet	0.96 (0.91–1.00)	1.00 (1.00–1.00)	0.93 (0.84–1.00)	0.99 (0.97–1.00)	0.394
Core wall	0.96 (0.91–1.00)	0.96 (0.89–1.00)	0.96 (0.89–1.00)	1.00 (0.99–1.00)	0.261
Core-wall wavelet	0.96 (0.91–1.00)	1.00 (1.00–1.00)	0.93 (0.84–1.00)	1.00 (0.99–1.00)	0.260

*Represents *p* < 0.05

### Comparison between the clinical and radiomics model

No significant differences were observed between the clinical model and all the radiomics models in the internal testing set (clinical vs. core radiomics: 0.93 vs. 1.00, *p* = 0.180, clinical vs. core-wavelet radiomics: 0.93 vs. 0.98, *p* = 0.358, clinical vs. wall radiomics: 0.93 vs. 0.77, *p* = 0.080, clinical vs. wall-wavelet radiomics: 0.93 vs. 1.00, *p* = 0.147, clinical vs. core-wall radiomics: 0.93 vs. 0.98, *p* = 0.397, clinical vs. core-wall-wavelet radiomics: 0.93 vs. 1.00, *p* = 0.147). In the external testing set, the clinical model outperformed the radiomics model based on the wall features (clinical vs. wall radiomics: 0.97 vs. 0.72, *p* < 0.001), but no significant differences were observed between the clinical model and all the other radiomics models (clinical vs. core radiomics: 0.97 vs. 0.98, *p* = 0.613, clinical vs. core-wavelet radiomics: 0.97 vs. 0.95, *p* = 0.687, clinical vs. wall-wavelet radiomics: 0.97 vs. 0.99, *p* = 0.394, clinical vs. core-wall radiomics: 0.97 vs. 1.00, *p* = 0.261, clinical vs. core-wall-wavelet radiomics: 0.97 vs. 1.00, *p* = 0.260).

## Discussion

In this research, we have identified 12 commonly observed clinical and imaging characteristics to develop a clinical prediction model. Additionally, we have extracted features from three groups including core, wall, and combined regions on DWI to establish six radiomics models using an automatic machine learning method. The objective of this study was to differentiate between cystic brain metastases and abscesses. Our findings indicate that both the clinical and radiomics models have achieved high AUCs. The optimal radiomics model demonstrated excellent predictive value in distinguishing cerebral cystic metastases from abscesses, with AUCs of 1.00 both in the internal and external test sets.

Previous research (Muccio et al. 2014) suggested that certain features of routine MRI sequences and clinical signs can aid in the differential diagnosis of cystic brain metastases and brain abscesses, and the DWI signal or ADC value has been particularly useful in increasing diagnostic effectiveness. However, related studies have shown varying sensitivities (64–100%) and specificities (77–100%) for DWI in this regard (Xu et al. 2014). Additionally, these researches have been limited by small sample sizes and a combination of few characteristics (Salice et al. 2016; Kolakshyapati et al. 2019; Schwartz et al. 2006; Alam et al. 2012). In this study, we incorporated 12 clinical and image features to build a clinical model. Our results showed that age, fever, leukocytosis, location, hypointense rims on T2WI, pattern of enhancement wall, degree of edema, ADC value of the core, ADC value of the wall, and maximum diameter of the mass were significantly different between the training and/or test sets (*p* < 0.05). The clinical model performed well, achieving an AUC of 0.93 in the internal test set and an AUC of 0.97 in the external test set. The larger sample size and increased number of characteristic combinations likely contributed to the improved performance of routine clinical data in distinguishing between cystic brain metastases and brain abscesses.

Some studies have shown that radiomics models based on DWI or ADC have higher values and benefits in differential diagnosis, evaluating biological factors, and predicting tumor prognosis (Xu et al. 2020; Park et al. 2020; Hu et al. 2022; Kim et al. 2022; Wang et al. 2021). In this study, we analyzed DWI as the single MRI sequence to build a radiomics model, which demonstrated superior diagnostic values for these two conditions. Our work showed that the DWI-based radiomics optimal model had AUCs of 1.00 both in the internal and external test sets, indicating its high efficiency in the differential diagnosis of cerebral cystic metastases and abscesses. This marks the first instance of radiomics being utilized for the differentiation of these two conditions. Unlike most radiomics research (Su et al. 2021; Priya et al. 2021; Li et al. 2022a, b), which manually conducted feature selection and chose trivial machine learning models, we used an automatic machine learning method for automatic feature selection, model selection, and parameter optimization. By intelligently exploring thousands of possible pipelines, TPOT automates the tedious part of machine learning and identifies the best pipeline (Le et al. 2020). In Wang’s study (Wang et al. 2023), TPOT was used to identify IDH‐mutant TERT promoter‐mutant gliomas, reaching an AUC of 0.952 in the independent validation set. In another study (Liu et al. 2022), TPOT was shown to differentiate brain metastases from glioblastoma with a higher AUC of 0.988 than using other classifiers. Our study, TPOT also showed excellent ability in differentiating cerebral cystic metastases from abscesses. Combining features from the cystic fluid core and solid wall improved the accuracy, sensitivity, specificity, and AUC. The top ten radiomics features were all first-order features, which describe the histogram distribution of voxel intensity in the image region. The features of mean, median, 90th percentile, and maximum mainly reflected the average and high voxel intensity. Skewness reflects the symmetric degree of data distribution. Root mean squared indicates the magnitude of image values. These parameters representing the density of the pathological lesion have the potential to quantify micro-architectural properties of tissues. Seven of the features were further processed using wavelet transforms, allowing for a comprehensive and accurate reflection of the original image. Although these features are difficult to identify with the naked eye, radiomics can make full use of them for disease identification. Significantly, the weight of wall_wavelet-HLL_firstorder_Mean extracted from solid wall were the largest among all the radiomics features. Meanwhile the wall-wavelet model also demonstrated excellent performance in the internal and external set. The result indicated that the DWI characteristics of solid wall, which might otherwise be overlooked in most studies of the two conditions, have offered added value to current radiomics study. Although there was no statistical difference between the optimal radiomics model and the clinical model, the radiomics model from a single DWI sequence yielded higher AUC value than the clinical model with many clinical features combined with multiple sequence MRI characteristics. In future studies, the inclusion of more heterogeneous group of cerebral metastases and abscesses in large samples will highlight the advantages of radiomics and may help to reach statistical differences.

Notably, MR images can exhibit significant variations depending on the scanning equipment, acquisition parameters, and inherent acquisition artifacts. These factors can cause instability in radiomics features (Cui and Yin 2022; Veres et al. 2022). Additionally, susceptibility artifacts and chemical displacement due to signal acquiring methods can impact the performance of DWI (Hu et al. 2022). Preprocessing methods can improve the reproducibility and stability of quantitative MRI analysis, leading to more reliable radiomics feature values (Moradmand et al. 2020). Our study utilized pyradiomics in Python to develop a preprocessing method that effectively reduces discrepancies in image data, resulting in improved robustness of feature extraction and model establishment.

The current study has some limitations. Firstly, it was conducted retrospectively and included a limited amount of data from only two medical centers. To enhance the generalizability and effectiveness of the model in clinical practice, it is recommended to conduct large-scale and prospective studies across multiple centers. Secondly, there was some selection bias in the retrospective study, as most patients with cerebral metastases had primary lung tumors, and only a small number had tumors originating from other sites, such as the esophagus, kidney, or colon. Additionally, most brain abscess cases were confirmed to be bacterial, with fungal and tubercular abscesses being rare. The predictive accuracy of the model may be affected by a more heterogeneous group of cerebral metastases and abscesses. Therefore, further research is needed to include more patients with these types of tumors.

## Conclusion

In summary, we have successfully constructed a high-performing radiomics model, utilizing automated machine learning techniques that can effectively differentiate cerebral cystic metastases from abscesses based on DWI. Furthermore, our preprocessing methodology has improved the dependability and durability of the initial results, which could greatly facilitate the practical applications of this model in clinical settings.

### Supplementary Information

Below is the link to the electronic supplementary material.

Supplementary file1 (DOCX 3947 KB)

## Data Availability

The datasets generated during and/or analyzed during the current study are available from the corresponding author on reasonable request.
