# Supplementary material for: Diffusion-weighted imaging-based radiomics model using automatic machine learning to differentiate cerebral cystic metastases from brain abscesses
Source: J Cancer Res Clin Oncol. 2024 Mar 16;150(3):132. doi: 10.1007/s00432-024-05642-4 (PMC10944436; doi:10.1007/s00432-024-05642-4)
Supplement: Supplementary file 1 — Supplementary file1 (DOCX 3947 KB) [file 432_2024_5642_MOESM1_ESM.docx]

**Supplemental Materials**

**Title: Diffusion weighted imaging-based radiomics model using automatic machine learning to differentiate** **cerebral cystic metastases from brain abscesses**

Supplemental Figure 1. The intra- and inter-observer ICC value. The value of intra- observer ICC were 0.96, The value of inter-observer ICCwere 0.95.

**
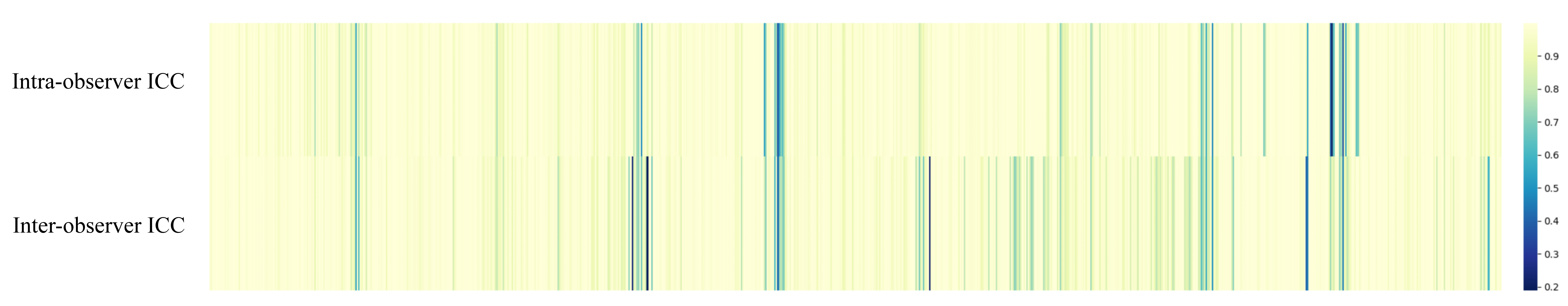
**
